# Fungal Mushrooms: A Natural Compound With Therapeutic Applications

**DOI:** 10.3389/fphar.2022.925387

**Published:** 2022-07-13

**Authors:** Rishi Man Chugh, Pooja Mittal, Namratha MP, Tanu Arora, Tanima Bhattacharya, Hitesh Chopra, Simona Cavalu, Rupesh K. Gautam

**Affiliations:** ^1^ Department of Radiation Oncology, University of Kansas Medical Center, Kansas, KS, United States; ^2^ School of Pharmaceutical Sciences, RIMT University, Mandi Gobindgarh, Punjab, India; ^3^ CHRIST (Deemed to be University), Bangalore, India; ^4^ Innovation, Incubation and Industry (i-cube) Laboratory, Techno India NJR Institute of Technology, Udaipur, India; ^5^ College of Chemistry and Chemical Engineering, Hubei University, Hubei, China; ^6^ Chitkara College of Pharmacy, Chitkara University, Rajpura, India; ^7^ Faculty of Medicine and Pharmacy, University of Oradea, Oradea, Romania; ^8^ MM School of Pharmacy, MM University, Sadopur-Ambala, India

**Keywords:** Medicinal mushrooms, immunomodulation, radical scavenging, antiviral, immune boosters, cardioprotective

## Abstract

Fungi are extremely diverse in terms of morphology, ecology, metabolism, and phylogeny. Approximately, 130 medicinal activities like antitumor, immunomodulation, antioxidant, radical scavenging, cardioprotective and antiviral actions are assumed to be produced by the various varieties of medicinal mushrooms. The polysaccharides, present in mushrooms like β-glucans, micronutrients, antioxidants like glycoproteins, triterpenoids, flavonoids, and ergosterols can help establish natural resistance against infections and toxins.. Clinical trials have been performed on mushrooms like *Agaricus blazei Murrill Kyowa* for their anticancer effect*, A. blazei Murrill* for its antihypertensive and cardioprotective effects, and some other mushrooms had also been evaluated for their neurological effects. The human evaluation dose studies had been also performed and the toxicity dose was evaluated from the literature for number of mushrooms. All the mushrooms were found to be safe at a dose of 2000 mg/kg but some with mild side effects. The safety and therapeutic effectiveness of the fungal mushrooms had shifted the interest of biotechnologists toward fungal nanobiotechnology as the drug delivery system due to the vast advantages of nanotechnology systems. In complement to the vital nutritional significance of medicinal mushrooms, numerous species have been identified as sources of bioactive chemicals. Moreover, there are unanswered queries regarding its safety, efficacy, critical issues that affect the future mushroom medicine development, that could jeopardize its usage in the twenty-first century.

## 1 Introduction

Fungi are extremely diverse in expression of morphology, ecology, metabolism, and phylogeny (Raja et al., 2017). Fungi produce a diversity of bioactive compounds, making them ideal for natural goods. About 130 therapeutic functions are believed to be formed by medicinal mushrooms and fungi, including antitumor (Deshmukh et al., 2018; Uzma et al., 2018) immunomodulating (Lull et al., 2005; Kanazawa et al., 2013)antioxidant (Sugiharto et al., 2016; Hameed et al., 2017), cardiovascular, anti parasitic, antiviral, antifungal, antibacterial, radical scavenging, hepato protective, detoxification, anti diabetic (Murugan et al., 2017), anti hypercholesterolemia as well as protection against tumor development and inflammation are exhibited by fungi. Polysaccharides, alkaloids, proteins, fats, minerals, carotenoids, glycosides, terpenoids, folates, tocopherols, flavonoids, phenolics, volatile oils, ascorbic acid, lectins, enzymes, and organic acids, are bioactive molecules synthesized by different fungal organisms.

A healthy, well-balanced diet is essential for illness prevention, especially when it comes to oxidative stress. Mushrooms have been utilized in oriental medication for centuries to prevent and treat a variety of diseases. Mushroom extracts are increasingly available as dietary supplements, especially to increase immune function and anticancer activities (Yuan et al., 2020; Kück et al., 2014). The mushrooms, or macro-fungi, are non-photosynthetic, eukaryotic, aerobic organisms that produce distinctive fruiting bodies. The mushrooms are divided into two groups based on their taxonomic classification: Basidiomycetes and Ascomycetes. More than 14,000 mushroom species have been identified, divided into 30 genera, and are now thought to be potential medicinal or preventative agents for humans, of which 2000 are edible. 270 species of edible mushrooms (Kumar et al., 2021) (*Neuronal Health–Can Culinary and Medicinal Mushrooms Help*)

In ancient times, the Mycophagy i.e., the mushrooms are being eaten. Edible species of mushrooms were found in Chile near the archeological sites, 13,000 years old back (Khatua et al., 2017). The edible mushrooms are being consumed for their gastronomic and nutritional, as well as gastronomic effects. Many fungal species are edible mushrooms, which are either picked wild or produced. The fleshy and eatable fruit bodies of several species of macrofungi (fungi with fruiting structures large enough to be seen with the naked eye) are edible mushrooms. They can grow above ground (epigeous) or, below ground (hypogeous) both of which can be harvested by hand. Markets frequently stock easily farmed and common wild mushrooms, while those that are more difficult to come by can be picked on a smaller scale by individual collectors. Many wild mushrooms are eaten throughout the world. *Agaricus arvensis*, *Amanita caesarea*, *Handkea utriformis, Cortinarius variicolor*, *Agaricus silvaticus*, *Ustilago maydis*, *Marasmius oreades*, *Leccinum versipelle*, *Suillus luteus,* etc. are some of the examples of edible mushrooms.

Mushrooms are prized in China for both their medicinal benefits and culinary. Medicinal mushrooms (MM) are used to prevent/heal or treat the no. diseases and also they maintain a healthy diet, MM as well as Fungus, are responsible for around 130 therapeutic activities. MM is rich in protein percentage content (20–30% dry matter) and is able to fulfill the need of all the amino acids. The mushrooms have no cholesterol and are low in total fat with a high amount of unsaturated fatty acids. They’ve been employed not only as a source of delectable meals and flavorings but also as a source of therapeutic ingredients. After the deep research on mushrooms, the therapeutic effects of the mushrooms were well understood. (Ganeshpurkar et al., 2010; Rahman et al., 2016). Medicinal mushrooms offer a variety of health benefits. They’ve long been used to improve health in civilizations all around the world, and new scientific research is beginning to keep these claims preserved (Mustafa et al., 2022). Numerous verities of mushrooms have been included in the data base of traditional medicines but, among them, the mushrooms which are highly beneficial includes: *Agaricus brasiliensis*, *Ganoderma lucidum*, *Trametes versicolor*, *Lentinus edodes*, *Flammulina velutipes*, *Agaricus bisporus*, *Tricholoma matsutake*, *Auricularia auricula*, *Pleurotus ostreatus*, *Grifola frondosa, Cordyceps sinensis*, *Coprinus comatus.* (Abdel-Azeem et al., 2019). Secondary metabolites, particularly low molecular weight chemicals like lactones, alkaloids, terpenoids, antibiotics with various chemical groups, and metal chelating agents, are other substances of therapeutic importance from the mushroom. These useful mushrooms have grown in popularity in the west over the last few years, are more widely available, and provide numerous benefits for our fast-paced modern lives. Mushrooms include adaptogens that help the body adapt to both internal and external stimuli, restoring equilibrium and regulating a variety of biological processes. The agriculture of mushrooms started long years back and now about twenty species are being cultivated. More the 60 countries are growing the mushrooms. Now, days, a small percentage of fungi is being cultivated and sold in the market as very few species are being consumed by humans. However, it is difficult to cultivate some of the species while for others, the satisfactory procedures for the cultivation has to be developed yet.

## 2 Mushrooms in Ancient Remedies

Ancient Romans and Greeks, most particularly the aristocracy, used to add mushrooms in their cookery. Even the Roman emperors used to hire the food tasters to ensure that the mushrooms are safer to eat. Farming of Mushrooms has the long history and currently, about twenty species are being commercially grown. They are cultivated in over 60 countries across the world. Only a small portion of the diverse fungus used by humans is currently grown and marketed commercially. In the past, Europeans were not the only ones who ate mushrooms. Since the Neolithic period, mushrooms have been utilized for medicinal purposes. The fungus Piptoporus betulinus, which has antibacterial properties and acts as a natural parasite killer (Kemami Wanguna et al., 2004; Pleszczyska et al., 2016), was discovered in the medicine box of the world’s oldest human mummy, which dates back 4,000 years. Mushrooms are pictured in Egyptian hieroglyphics as the immortality plant, known as the “sons of the gods,” which was brought to Earth on lightning bolts and was only consumed by aristocracy and pharaohs. Sacred mushrooms known as “the meal of the gods” were also eaten by the Aztecs during religious rites.

Special mushrooms, particularly the Reishi fungi, were prized as a tonic herb in ancient China and were restricted to the general people. Buddhist monks traveling from monastery to monastery disseminated knowledge about the healing properties of fungi, which they and Taoist priests employed in rituals. The Buddha is said to have died after eating a toxic fungus! This psychedelic is now available in a contemporary form. Before the battle, the Vikings are thought to have used LSD (lysergic acid diethylamide), which contains psychedelic mushrooms, which would have resulted in the vicious fighting state for which they are known. Europeans may have used magic mushrooms to improve religious rites as long as 6,000 years ago. According to a cave painting in Spain that may depict hallucinogenic fungi, this is the case. Authors such as Pliny, Seneca, and Dioscorides–author of De Materia Medica—argued both for and against mushrooms as medicine in ancient Greece and Rome. The hallucinogenic potion Kykeon, an ergot-barley-and-mint drink, was consumed by Socrates, Plato, and other ancient Greek elites during the Eleusis festival, which celebrated birth, regeneration, and the return of spring.

## 3 Therapeutic Efficacy of Different Mushrooms

Our best line of defense against illness is a healthy immune system, which is also the key to a preventative healthcare approach to general long-term wellness. These mushrooms are regarded to be powerful immune-boosting superfoods (Valverde et al., 2015). Polysaccharides, like as β-glucans, micronutrients, antioxidants like glycoproteins, triterpenoids, flavonoids, and ergosterols can help establish natural resistance against infections and toxins. They were widely used as nutritional supplements, including mushrooms at various stages of development. Mushrooms’ functional qualities are determined by their bioactive compounds. To combat chronic diseases linked to oxidative stress, bioactive compounds found in mushrooms can be removed and purified for usage as nutraceuticals or in functional meals (Valverde et al., 2015). There are a variety of edible mushrooms that have therapeutic efficacy.

3.1 *Agaricus subrufescens* is known as “almond mushroom” because of the almond flavor. This is farmed in the United States and has been mistakenly identified as *Agaricus blazei*. It produces a variety of bioactive substances that have the ability to treat a variety of ailments, and it has been utilized as a medicinal food for cancer, diabetes, hyperlipidemia, hepatitis, arteriosclerosis, and prevention. Tumor growth inhibition, antibacterial and antiviral activity and immunostimulatory and antiallergy actions are only a few of its therapeutic qualities. Polysaccharides such as riboglucans, β-glucans, glucomannans make up a majority of the bioactive chemicals extracted from this mushroom. Ergosterol, a lipid component, has been discovered to have anticancer activity (Firenzuoli et al., 2008; Wisitrassameewong et al., 2012; Takaku et al., 2001).

3.2 The “Cinder Conk” or the “Chaga Fungus” are produced in cold climates on the birch trees all throughout the world and are being utilized mostly by Russian herbalists as a treatment to restore vitality and maintain health (Kang et al., 2015). The mushroom includes a variety of antioxidants, including superoxide dismutase, melanin, and triterpenes like lupeol, inotodiol, and betulin, in addition to polyphenols, sterols, and polysaccharides. Betulinic acid, which is found in large proportions in birch bark, is one of its primary components. The crusty charcoal-like coating of Inonotus obliquus is also a natural source of melanin. (Balandaykin and Zmitrovich, 2015).

3.3 Maitake, commonly known as “signorina,” or “hen of the woods” is a natural recurrent fungus that develops in the same spot year after year, generally near oak trees. In recent years, it has also been grown to be marketed fresh, dried, or prepared as a supplement. DFraction, a unique protein-bound beta-glucan, is one of its primary polysaccharides (Ulbricht et al., 2009). One of the key components attributed to maitake’s potent immune-modulating properties is this micronutrient. Maitake is often cooked as a fresh type and used as a “gourmet mushroom” by chefs around the world, delivering a flavorful taste in soups, sauces, and a variety of cuisines.

3.4 Cordyceps sinensis supports adrenal function. It is also known as nourishment for the lungs and kidneys. It possesses the aphrodisiac properties for both women’s and men’s sexual organs (Shashidhar et al., 2013). β-glucans and CO-1 are found in *Cordyceps*, as well as a chemical termed “cordycepin,” which is specific to this mushroom type. It is known as a “Chi”-building plant with capabilities that are supposed to “activate life forces,” and its medicinal benefits are often compared to ginseng. C. sinensis is commonly used as an athletic support tonic for recovering physical stamina and enhancing performance. (Ulbricht et al., 2009).

3.5 Trametes versicolor (Coriolus) is a well-known mushroom species which is found in a number of temperate climates around the world. Because it forms vast shelf-like clusters on rotting trees and logs, it is simple to recognize. Because of its shape, lines, and colors, which are reminiscent of wild turkey tail feathers, it is known as “turkey tail.” This is amongst the most studied medicinal mushrooms. Two polysaccharides, kresin (PSK) and polysaccharide peptide are the main beta-glucan-rich constituents of Coriolus or turkey tail (PSP). “The cloud mushroom” is another name for it. The best and most medicinally effective approach to use this fungus is as a prepared liquid or powdered extract.

3.6 Agaricus blazei is a Brazilian mushroom species that is likewise known as “cogumelo do sol” (Sun mushroom) and *Agaricus brasiliensis*. It is a different sort of mushroom that may be eaten fresh and has a sweet almond-like flavor. To unleash its helpful healing components, it should be treated with hot water or an alcoholic solution.

*Agaricus* contains a significant number of beta-glucans, which aid in immune function modulation. It is linked to the white button *Agaricus bisporus* mushroom, which is commonly known as cremini or portobella (Blumfield et al., 2020). According to the research, the white mushrooms which are available on the market are devoid of many medicinal characteristics. In Europe and North America, in a variety of varieties, Agaricus bisporus dominates the edible mushroom market. It is a Basidiomycetes fungus that thrives in European and North American meadows. As it ages in time, this mushroom matures from a small white smooth mushroom to a giant light brown mushroom. It is also called the “button mushroom” (FUNGI USED AS FOOD History of Mushroom Use, 2020).

3.7 Ganoderma lingzhi (Reishi) has been documented in Asian culture’s earliest pharmacopeia writings stretching back thousands of years. Because of its relaxing effect on the nerves and kidney system, it is regarded as a powerful “Shen” tonic. It is said to “open the spirit” in Traditional Chinese Medicine, and Taoists have employed it to achieve Enlightenment or “spiritual immortality.” Ganoderma lucidum is another variety of mushrooms which aids neuron renewal and treats insomnia, that’s why it is considered to be a great supplement for a good sleep. The mycelium of the reishi mushroom contains peptides, nucleotides, sterols, polysaccharides, Triterpenoids, steroids, and numerous trace elements. (Cunha Zied & Pardo-Giménez, 2017; Abdel-Azeem et al., 2019).

3.8 Lentinula edodes (Shiitake) Because of their rich flavor and meaty texture, shiitake mushrooms are one of the most popular edible “gourmet” mushrooms. However, many people are unaware that shiitake mushrooms are also extremely medicinal, carrying a number of therapeutic compounds. It is made composed of beta-glucans and other polysaccharides like lentinan, emitanin, and KS-2, and it is been intensively researched for its potential pharmacological characteristics. (He et al., 2017). It is also widely used as a nutritional supplement or a hot water extract, which is normally prepared from the whole mushroom, mycelium biomass, or separated bioactive components, including lentinan extracts, for increased therapeutic potency(Ganeshpurkar et al., 2010).

3.9 Lion’s name is well known for the nervous system and brain (Kang et al., 2015; He et al., 2017). It got its popularity as a most commonly used therapeutic variant as it acts like a nootropic and neurotrophic superfood. In research, it has been proven to be very effective since 1990 in the stimulation of the growth of nerve growth factors and it secretes the proteins which play an important part in the conservation, regeneration, and survival of the neurons. Two distinct chemicals namely hericenones and erinacines are present in this which makes it suitable to be used as a “nootropic” dietary supplement to boost cognitive processes. Other studies also support its use in the treatment of dementia and illnesses like Parkinson’s and Alzheimer’s disease. (He et al., 2017).

## 4 Immunoregulatory Compounds and Industrial Products of Medicinal Mushrooms

They are the different varieties of mushrooms with different taxonomy. Some of these mushrooms are being cultivated for a variety of foods also, but due to their immunomodulatory effects, these mushrooms are increasingly becoming part of nutraceuticals. The important immunomodulatory compounds found in medicinal mushrooms are terpenes, flavonoids, terpenoids, lectins, polysaccharides particularly D-glucans and fungal immunomodulatory proteins, etc. (Enshasy and Hatti-Kaul, 2013).

### 4.1 Polysaccharides

In the last decades, polysaccharides which include both polysaccharopeptides and polysaccharide proteins which can be with or without the side chains are among the utmost studied medicinal mushrooms for their immunomodulatory actions. Lentinan, derived from shiitake (*Lentinus edodes*), and schizophyllan, derived from the *Schizophyllum commune*, are the most well-known polysaccharides which are obtained from *Schizophyllum commune* posses immunomodulatory as well as anti-cancer properties. Both schizophyllan and lectinan include 1,3-D-glucans with 1,6 branches (Sindhu et al., 2021; Rahman et al., 2022a). Among these, Lantinan was found to possess the immunomodulation properties against stomach cancer while schizophyllan was found to posses the anticancer properties against head and neck cancer. (Hosseini et al., 2016; Zhao et al., 2020). In 1986, both of these drugs were licensed to be used in Japan for their chemotherapeutic purpose. Other polysaccharide compounds that possess a similar base structure with different branches, also possess immunomodulatory properties. Heteroglucans are polysaccharideconjugate complexes with immunomodulatory properties. (Zhao et al., 2020). Other compounds that have been shown to possess immunomodulatory action are *Agaricus brazei* and *Macrocybe gigantea*. A polysaccharide-protein complex (PSPC) screened from a *T. giganteum* was found to help in the improvement and repairment of the macrophage phagocytic function in tumor-bearing mice. (Zhao et al., 2020; Moradali et al., 2007). AbM from the *A. brazei* also comprises polysaccharide-protein chain complexes with diverse chemical bond linkages, including a 6glucan, 1,6- and 1,4-glucan, glucomannan, and 1,3-glucan. AbM is found to possess anticancer and immunomodulatory properties. (*Ooi 2001*, n. d.). Table 1 presents few mushrooms derived polysaccharides and their protein complexes for immunomodulatory actions.

**TABLE 1:** Various medicinal mushrooms derived Polysaccharides and polysaccharide-protein complexes which are immunomodulatory.

Source	Active compound	Immunomodulatory effect	References
*Auricularia auriculajudae*	3-D-glucan, AF1 β-1 main chain with the two β-1,6-Dglucosyl residues	Produces cancer cell apoptosis	Ma et al. (2010)
*Flammulina Velutipes*	The glycoprotein, Flammulina velutipes peptidoglycan (FVP), β1,3-D-glucan	Secrete Tumor necrosis factor alpha (TNF-α), Interleukin production	Yin et al. (2010)
*Lentinus squarrosulus*	Glucan	Induces the splenocytes and thymocytes	Bhunia et al. (2011)
*Sparassis Crispa*	β-Glucan	Promote Interferon gamma (IFN-γ) and Interleukin-6 (IL-6) synthesis	Bimczok et al. (2009)
*Tremella fuciformis*	Acidic glucuronoxylomannan of an α-1,3-D-mannan with d-glucuronic acid	Human monocytes synthesized	Wasser, (2002)

### 4.2 Protein–Conjugate Complexes and Mushroom Proteins

In terms of immunomodulatory chemicals, medicinal proteins produced by mushroom and their conjugate-protein complexes are popular. These protein-based substances which have an immunomodulatory effect in medicinal mushrooms can be classified in the same way that polysaccharide-based compounds can. These substances are classified into two groups-lectins and fungal immunomodulatory proteins (FIPs) (Money, 2016). FIPs vary from lectins in that they lack conjugate, whereas lectins have particular carbohydrates attached to polypeptides. Table 2 shows a few of the lectins present in medicinal mushrooms that are known to have immunomodulatory properties. These lectins are demonstrated to stimulate nitrite synthesis, upregulate the expression of TNF-α and activate lymphocytes, and interleukins, and promote the initiation of macrophage-activating factors, among other things.

**TABLE 2:** The immunoregulatory effects of lectins found in medicinal mushrooms.

Source	Lectin Name	Immunomodulatory Effect	References
*Agrocybe aegerita*	Agrocybe aegerita lectin (AAL)	Inhibits the proliferation of HL60 SW480, 4T1, HeLa, MGC803, BGC823, SGC7901, and S180 cells	Zhao et al. (2020)
*Boletus edulis*	Boletus edulis lectin (BEL)	Induces splenocyte mitogenicity in mice and inhibits the growth of human hepatocyte carcinoma HT-29 and G2 cells	Zheng et al. (2007)
*Grifola frondose*	Grifola frondosa lectin (GFL)	Inhibits the HeLa acceleration	Kawagishi et al. (1990)
*Russula lepida*	Russula lepida lectin (RLL)	Inhibits the build-up of 2 MCF7 cells and HepG	Zhang et al. (2010)
*Xylaria hypoxylon*	Xylaria hypoxylon lectin (XHL	Inhibits the propagation of HepG2 cells	Liu et al. (2006)

The medicinal mushroom species in therapeutic lectins are many (Zhao et al., 2020). Table 3 represents the immunoregulatory effects of FIPs found in medicinal mushrooms.

**TABLE 3:** The immunoregulatory effects of FIPs found in medicinal mushrooms.

FIP Name	Source	Immunomodulatory effect	References
FIPcru1	*Chroogomphis rutilus*	Vital growth of the murine splenocytes increases secretion of IL-2	Liu et al. (2006)
FIP-gmi	*Ganoderma microsporum*	Controls the TNF-α	Lin et al. (2010)

Additionally, to immunomodulation, many FIPs have been demonstrated to exhibit anticancer properties in pharmacological studies, such as inhibiting proliferation and cell growth, inducing autophagy and apoptosis, and reducing tumor cell invasion and migration. Currently, tissue cultures are used in the majority of these studies. To establish their safety and efficacy in humans, more research utilizing animal model studies and clinical trials are required. If validated, these FIPs could be more synthesized efficiently and marketed for clinical administration thanks to genetic engineering (Smith and Sullivan, 2002).

### 4.3 Terpenoids and Terpenes

**FIGURE 1:**
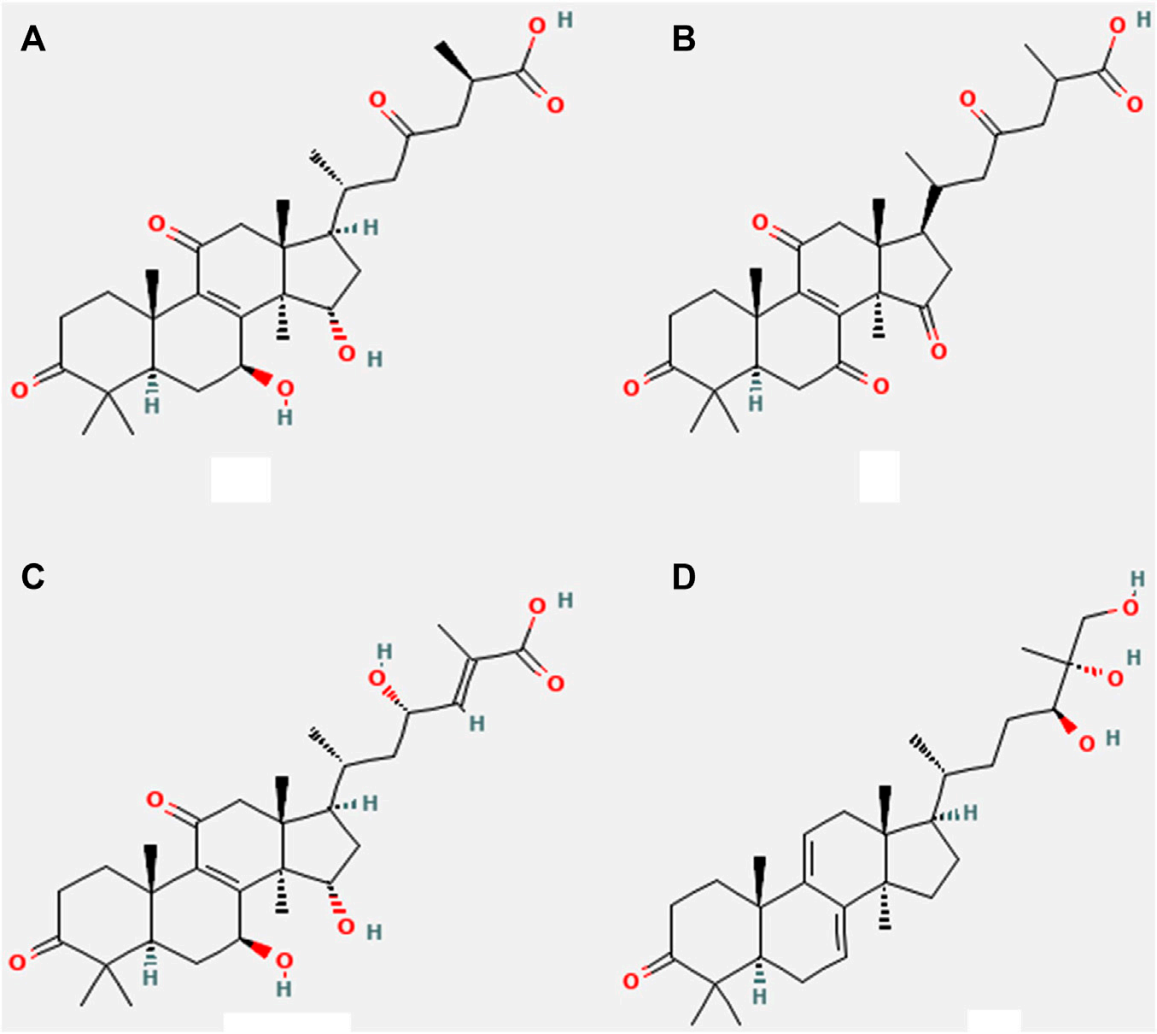
Various triterpenoids derivatives **(A)** Ganoderic acid A300 **(B)** Ganoderic acid E300 **(C)** Ganoderic acid gamma 300 **(D)** Ganodermanontriol 300.

Terpenes and terpenoids can be found in abundance in mushrooms. They are a diverse set of organic compounds that share a common center of isoprene five-carbon atom units with its chemical formula (C_5_H_6_) n in the primary building block (Figure 1). The triterpenoids from *G. lingzhi and G. lucidum* are probably the most well-known of this category of chemicals. These triterpenoids can aid to prevent nephrotoxicity and inflammation caused by drugs. In *G. lingzhi* and *G. lucidum* a variety of terpene derivatives, including ganoderal, ganodermic, ganoderic acids, lucidone, ganodermanondiol, ganodermanontriol are shown in Figure 1. All of these drugs have anticancer, immunomodulatory, and/or anti-infective properties (Jeong et al., 2008). Their mode of action, as well as the structure-activity connections, are yet unknown (Wang et al., 2013;K. Ma et al., 2014). Their diverse actions, on the other hand, propose that they have a lot of potential for clinical treatment and research uses (Sohretoglu and Huang, 2018).

## 5 The Cytostatic Function of a Medicinal Mushroom

Mushrooms, both edible and medicinal, can produce a wide range of physiologically active chemicals, making them a distinct class of nutraceuticals that are frequently used as dietary supplements. Although mushrooms, their extracts, and separated metabolites are not medications, they are a sort of significant nutritional supplements, such as nutraceuticals or functional food, and could be employed as naturally available cytostatics as well. Recent epidemiological research from Asia has shown that mushroom consumption reduces the risk of cancer. *Ganoderma* species which are well known for their capacity to produce different variety of compounds with intriguing biological effects. *G. applanatum* (Jeong et al., 2008) and (Osińska-Jaroszuk et al., 2014)fruiting bodies are frequently employed in traditional Chinese medicinal treatments (Lee et al., 2011). They are known for their anticancer, immunostimulatory, and antiviral properties. Biologically active compounds derived from *G. applanatum*, according to available studies, can be utilized to cure cancer and have a therapeutic effect against HIV (Awadasseid et al., 2017). The biological functions of selected mushroom extracts are given in Table 4.

**TABLE 4:** Biological activities of various mushroom extracts and mechanism of action.

Extract Sources	Target Cancer Cells	Mode of Action	References
*Agaricus bisporus*	Breast	Inhibits the stimulation of caspase-3 and aromatase activity	Adams et al. (2008)
*Agaricus sylvaticus*	Colon	Stimulates the immune system	Fortes et al. (2009)
*Agaricus blazei*	Uterus, leukemia, ovary, prostate	Inhibition of the telomerase activity, initiates the cytochrome c release, caspase activity, control of Bcl-2 synthesis	DeSouuza et al. (2017)
*Coprinus comatus*	Hepatoma and breast	Inhibits the NF-κB factor	Chen et al. (2007)
*Cordyceps militaris*	Hepatoma, leukemia, breast	Initiation of IFN-γ,IL-18	Patel et al. (2012)
*Cordyceps sinensis*	Hepatoma and Colon	Suppress the NF-κB factor	Patel et al. (2012)
*Flammulina velutipes*	Breast, colon cervix	Arresting cell cycle	Ayeka, (2018)
*Ganoderma lucidum*	Cervix, colon, prostate adenocarcinoma, lung, stomach	Stimulates the TNF- γ, caspase activation, inhibits the matrix metalloproteinase expression, suppression of transcription factor AP-1	Gill et al. (2016)
*Hericium erinaceus*	Colon	Activates the macrophages, natural killer cells, and angiogenesis inhibition	Kim et al. (2011)
*Lenzites betulinus*	Cervix, colon	Antioxidative	Milovanovic et al. (2015)
*Phellinus linteus*	Bladder, breast, liver hepatoma,, stomach lung	Induces IL-12, IFN-γ, synthesis of TNF-α, T- and macrophage, CD^4+^-, the proliferation of the dendritic cell, activation of a natural killer cell, an increase of thymus mass and plasma immunoglobulin receptors, spleen, reduces the epidermal growth factor receptors	Stajic et al. (2019)
*Pleurotus eryngii*	Cervix, colon	Antioxidative activity	Ma et al. (2017)
*Pleurotus ostreatus*	Cervix, colon	Increases the cytokines, antioxidative activity	Stajic et al. (2019)
*Pleurotus pulmonarius*	Cervix, hepatoma	Antioxidative activity	Khan et al. (2012)
*Trametes versicolor*	Cervix, colon, melanoma, leukemia, lung, lymphoma, prostate, stomach	Antioxidative activity, antimutagenic activity	Stajic et al. (2019)

## 6 Edible Mushrooms in Neuro Nutraceuticals and Cardioprotective Effect

Medicinal mushrooms are of higher-class fungi with various nutraceutical properties such as low-fat content, high fiber content, and a trans isomer of unsaturated fatty acids, biologically active compounds like polysaccharides, alkaloids, steroids, polyphenols, polysaccharideglucans, terpenoids, and alkaloids, steroids, polyphenols, and terpenoids (Badalyan et al., 2019). *In vitro* tests, animal model study, and even human study trials have shown that mushroom extract, as well as fresh edible mushrooms, have a huge range of therapeutic applications in human health benefits, including anti-diabetic qualities, cardioprotective, and antiobesity, (Khatun et al., 2012). Many mushrooms have been identified which correspond to the amelioration of CVD aetiological components, and its main active metabolites have been thoroughly investigated (Ely and Berne, 1992). Due to its fiber content, microelement content, protein, and mushrooms, notably *L. edodes*, *G. frondosa*, and *P. ostreatus*, are mostly ideal for low-fat calorie diets to avoid CVD to maintain a healthy lifestyle. Many bioactive compounds which are extracted from *Boletus aestivalis*, *G. frondosa*, *L. edodes*, *G. lucidum*, *Clitocybe nuda*, *H. marmoreus*, and *Pleurotus* species helps to maintain the levels of low, total cholesterol, high-density homocysteine, and lipoproteins to prevent the development of arterial oxidative stress, hypertension, and cardiovascular disease (Khatun et al., 2012).

**FIGURE 2:**
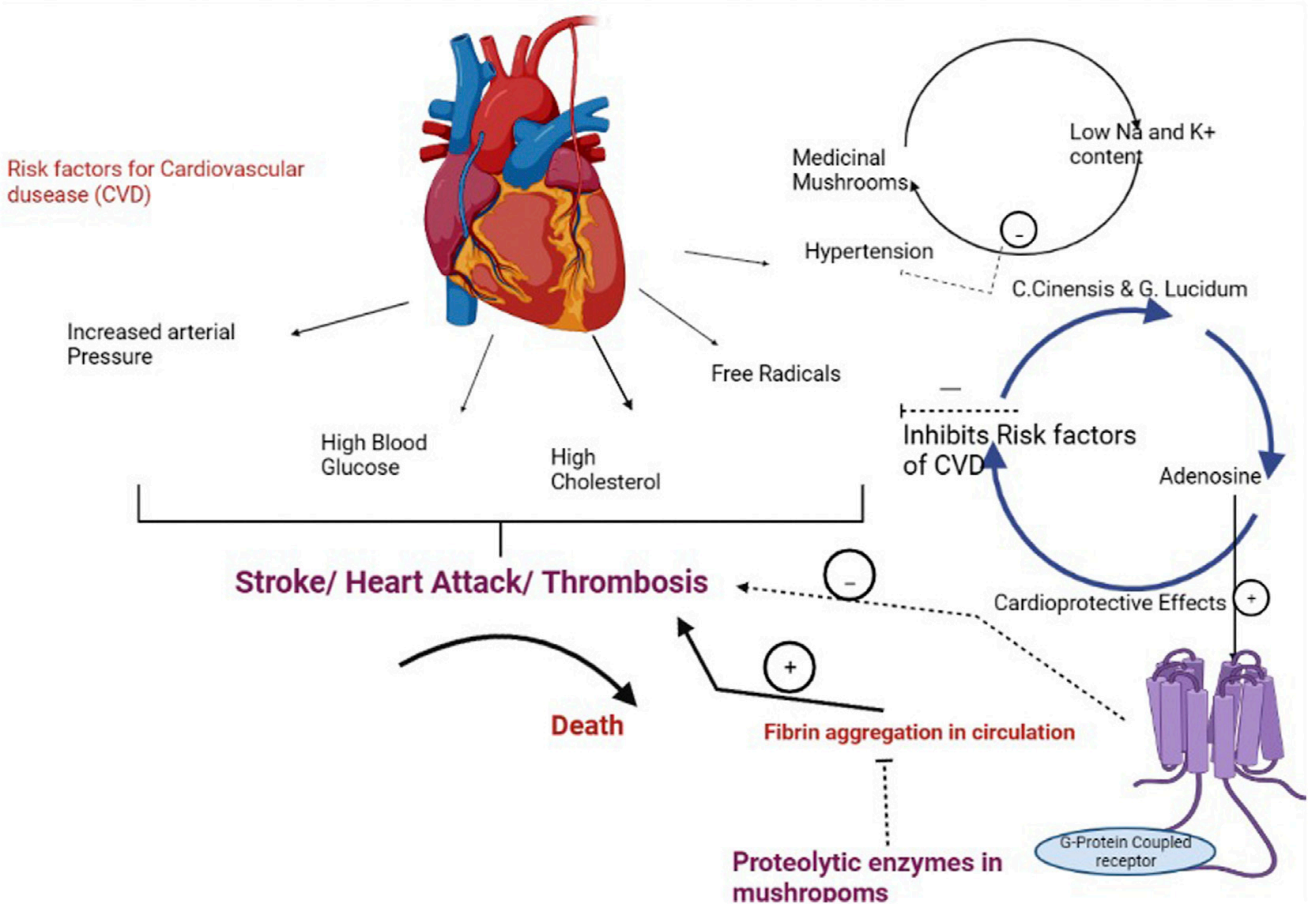
Pathway depicting the role of medicinal mushrooms in prevention of cardiovascular diseases.

Cardiovascular disorders (CVDs), which include heart attacks and stroke, hamper the circulatory system, and heart disease is the leading cause of death globally. Elevated artery pressure, high blood glucose, and high cholesterol are the key risk factors for cardiovascular disorder (CVD) (Badalyan et al., 2019). Different medicinal mushrooms have hypocholesterolemic and hypoglycemic qualities, allowing them to be used as a natural, healthy meal to prevent disease and promote cardiovascular health. Many medicinal mushrooms, such as *Cordyceps sinensis* and *Ganoderma lucidum*, contain adenosine, which has long been known for its cardioprotective properties. It binds to certain receptors that are linked to G-proteins, as well as to different effector systems. Endogenous and exogenous adenosines are both implicated in myocardial ischemia protection, which protects the heart from the harmful consequences of insufficient blood flow and oxygen supply, according to recent research. Due to fibrin aggregation in the circulation, thrombosis can potentially induce CVD (Previtali et al., 2011). The pathway depicting the role of medicinal mushrooms in cardiovascular diseases in presented in Figure 2.

Proteolytic (fibrinolytic, thrombolytic, caseinolytic, and other) enzymes (Lu and Chen, 2012) are active producers in mushrooms from many taxonomic and ecological groupings (Choi et al., 2013). *F. velutipes* was the first to identify proteases having fibrinolytic activity. Few Basidiomycetous mushrooms were then tested for thrombolytic and fibrinolytic properties. Fibrinolytic proteases is detected in mycelia and the fruiting bodies of medicinal mushrooms such as *Armillariella mellea*, *Auricularia polytricha*, and coprini species in several systematic studies. Hypertension, or high blood pressure, produces a lot of stress and harms heart function. Mushrooms are good dietary supplements to reduce hypertension since they have low sodium content and high potassium content (182–395 mg/100 g). Various studies have revealed that mushrooms including *Lentinula edodes*, *Pleurotus narbonensis*, (Inacio et al., 2015), *Ganoderma lucidum and Grifola frondosa* have antihypertensive properties (Ely and Berne, 1992). Table 5 represents the various bioactive compounds having anti hypertensive properties obtained from fungal mushrooms. Antioxidant consumption is an effective method for preventing the emergence of certain CVD problems. Mushroom polysaccharides and phenolic compounds have potent antioxidative effects. They significantly neutralize the radicals by increasing the activities of oxidative enzymes including catalase, glutathione peroxidase, and superoxide dismutase, as well as stabilizing malondialdehyde and glutathione levels. Antioxidant mushrooms include *Ganoderma lucidum*, (Kumaran et al., 2011), T*. mummiformis Coriolus versicolor G. Lentinula edodes tsugae*, and, Termitomyces heimii.

**TABLE 5:** Antihypertensive mechanism and bio components from the edible mushrooms.

Component	Mode of Mechanism	Source	References
Tripeptide 3,3,5,5tetramethyl- 4piperidone (TMP)	Partial ganglionic blocking mediated path	*Marasmius androsaceus*	Yahaya et al. (2014)
	Vasodilation	*Tricholoma giganteum*	Yahaya et al. (2014)
D-mannitol	Inhibits the ACE rapidly	*Pleurotus cornucopiae*	Abidin et al. (2017)
Oligopeptides	Inhibits the ACE rapidly	*Pleurotus cornucopiae*	Correa et al. (2016)
Potassium	Hyperpolarizes the Kir channels and/or Na + −pump	*Lentinula edodes*	Yahaya et al. (2014)
Lentinan	Vasodilation	*Lentinula edodes*	Yahaya et al. (2014); Yong et al. (2022)
Pentapeptide	Competitive and stereoselective	*Pholiota adiposa*	Yahaya et al. (2014)
l-pipecolic acid	Inhibition of ACE	*Sarcodona spratus*	Yahaya et al. (2014); Shibu et al. (2017)
Hexapeptide	Inhibition of ACE	*Grifola frondosa*	Choi et al. (2001)
Maleic/succinic acid derivatives, triterpenoids, benzenoids, and benzoquinone derivatives	Reduces the growth and phosphorylation of PKC in phorbol- 12,13dibutyrate (PDBu)activated platelets	*Antrodia camphorata*	Shibu et al. (2017)
Oligo peptides, protein extract	Inhibition of ACE	*Agaricus bisporus*	Lau et al. (2014)

Atherosclerosis is a chronic illness characterized by the secretion of lipids and fibrous materials in big blood arteries, it is the main risk factor for cardiovascular disease. Various edible mushrooms, as well as the mode of action responsible for the antiatherosclerotic activity, is been extensively explored in this regard (Badalyan et al., 2019). Some of the promising anti-inflammatory mushrooms are *Grifola frondosa*, *Pleurotus forida* and *Hypsizygus marmoreus*.

## 7 Recent Process and Clinical Applications

Unsaturated fatty acids, phenolic compounds, triterpenes, glycoproteins, and peptides are among the bioactive elements found in mushrooms, which are the fruiting bodies of macrofungi. The more abundant β-glucan polysaccharide seen in fungal cell walls is β-1, 3– glucan, which accounts for 65–95% of total β-glucan content. Β-glucans have a wide range of structural properties, which could affect how the body reacts to them, both in terms of protection against fungal infections and in terms of therapeutic immune activation. CR3 is likewise bound by β-glucans. CR3 may be the major β -glucan receptor on neutrophils and natural killer cells (NK) (Goodridge et al., 2009), it governs fungal death and phagocytosis in a complement-dependent way. Β-Glucans can also be fermented with the help of gut bacteria, which could result in favorable changes in the microbiome of the host (Goodridge et al., 2009) and (Choi et al., 2013). Numerous mushrooms have been demonstrated to be potent immune stimulators and may have an impact on a range of immunological targets (Guggenheim et al., 2014). These and many other medicinal mushrooms may have substantial therapeutic promise to persons with acute or chronic viral infections due to their immune-supporting qualities (Shahzad et al., 2020). A proprietary supplement containing a combination of curative mushrooms (including *T. Versicolor*, *G. lucidum*, *Cordyceps militaris*, *Agaricus blazei*, *Cordyceps Sinensis*, *Grifola frondosa14rondose*, and *Lentinula edodes*, with definite amounts of each not provided due to the proprietary nature of the formulation) was found to notably increases CD4^+^ T lymphocyte counts in adults with HIV who were not taking antiretroviral medication (Shahzad et al., 2020). Several fascinating research on the benefits of curative mushrooms in cancer patients has been conducted. Many of the benefits reported in oncology can be due to mechanisms such as modification of cellular and humoral immunity, meanwhile, others can be attributed to an unbroken antitumor effect (Lucius, 2020). Patients with breast, prostate, colorectal, hepatic, and lung malignancies, among others, have participated in mushroom clinical trials (Lucius, 2020). The immunomodulatory effective nature formed on immune cells may be determined by the manner of extraction of medicinal mushrooms, resulting in the varied solubility of inhibitory mediators and stimulatory (Hetland et al., 2021).

### 7.1 The Significance of Fungus in Drug Discovery and Development

With respect to Alexander Fleming’s serendipitous Penicillin discovery from Penicillium notatum, antibiotics from microbes became widely studied in academia and industry. This marked the start of the antibiotic golden age, which lasted more than 60 years (Panda et al., 2022). Traditional phenotypic screening, on the other hand, has been superseded by more rational and intelligent ways of exploring bioactive chemicals from fungus and microbes. Fungi have been majorly responsible for developing various bioactive substances with their wide properties, including Cyclosporine, Caspofungin, Lovastatin, and Fingolimod, among others. Natural goods, inspired or mimicked, have their origins in fungi and account for around 40% of the 1453 New Chemical Entities (NCEs) accepted by the USFDA. As a result, fungal compounds account for a critical role in the pharmaceutical industry’s drug discovery and development. Mushrooms’ diversity, ease of cultivation, and growing popularity make them one of nature’s best gifts for finding new natural goods, including medications (Plácido et al., 2022).

Nevertheless, only selected mushrooms and their simplified molecules have been studied clinically, despite the fact that they have already shown triggering or hindrance factors of particular cancer-related reactions, such as activation or inhibition of NF-κB, inhibition of proteins, mainly aromatases, tyrosine kinases, sulfatases, matrix metalloproteinases, DNA polymerase, cyclooxygenases, and DNA topoisomerases.

Future clinical trials should focus on evaluating the effectiveness of a big number of lowmolecular-weight drugs in appropriate dosages, either alone or in conjunction with existing anti-cancer treatments. Currently, evidence of mushrooms as anticancer usage is partial, and the scientific and analytical procedural quality of a few clinical research may be better (Panda et al., 2022). Although multiple articles have proven *in vitro* activity, the available information in many investigations only allows for preliminary judgments. Interestingly, various *in vitro* investigations related to mechanisms of action have specifically proven effects of immunomodulatory such as lymphocyte proliferation and changes in immunoglobulins and cytokines, among other things.

Although progress has been made in mouse tumor models, clinical trials are still limited. The reliability and validity of those investigations are harmed by an inconsistency in the preparation of procedures, bulky patient sample numbers, modes of administration, and longlasting follow-up studies Cavalu et al., 2018). More research is required to support the function of mushrooms in cancer prevention and management, in addition to their inclusion in a healthy diet. As more people utilize mushrooms as a comedication, there is an immediate cause to investigate the safety and efficacy of MM in welldesigned RCTs. MM has the potential to improve and analyze QOL both during and after traditional cancer treatment. (Cavalu et al., 2020).

To better understand cancer biology, a variety of early screening and detection strategies have been used, as well as a variety of tools and techniques to separate bioactive molecules from natural resources (Cui et al., 2020). The most demotivating aspects for pharma companies to explore natural product-based therapy include the difficult drug development process and validation status, as well as more failure rates in the translational phase. Several issues, such as medication delivery, toxicity, and pharmacokinetics profile, cause almost over 90% of drug research scholars to fail in the clinical translation stage (Rahman et al., 2022b). To persuade a pharmaceutical company to begin clinical development trials, significant experimental proof in the preclinical stage is required, bioactive molecules could be isolated through bioassay-guided purification, technique S. et al., 2022). To better understand cancer biology, a variety of early screening and detection strategies have been used, as well as a variety of tools and techniques to separate bioactive molecules from natural resources have been introduced. (Markham et al., 2020); and (Loud and Murphy, 2017).

## 8 Mushroom Pharmacological Activities: Medical Evidence From Clinical Trials

Few fungal metabolites or extracts have been demonstrated that are useful for *in vitro* tests or preclinical investigation, due to their potential remedial significance it been tested *in-vivo* in human patients in clinical trials.

### 8.1 Medicinal Mushrooms in Cancer Clinical Studies

• A random (RCT) clinical study of 100 patients who had trouble from many gynecological cancers (ovarian, cervical, and endometrial) and undergoing chemotherapy employed an extract of *Agaricus blazei Kyowa*, which is observed to have antimutagenic and anticancer characteristics (Ahn et al., 2004). The treated group had higher NK cell activity, but no variance in monocyte activity, and the lymphokine-activated killer was identified. Additionally, administration of the fungal extraction reduced chemotherapy-allied adverse effects such as loss of emotional imbalance, appetite, baldness, overall weakness, and a significant enhancement in mood-concerned parameters (anxiety, mental stability, and depression).
• Ohno et al. (Ohno et al., 2011) undertook the phase I clinical experiment to regulate the safety and efficacy of an A. blazei Murrill therapy for cancer-related patients. For 6 months, seventy-eight cancer patients (30/24/24) were administered Senseiro (1800 mg/pack) in one, two, or three packs. According to the National Cancer Institute (NCT), Common Terminology Criteria for Adverse Events version 3.0, adverse events were fixed by objective/subjective symptoms and laboratory data (NCI-CTCAE v3.0). The medicinal mushroom was shown to be harmless in virtually all of the patients. Only nine incidences of adverse effects, mostly digestive such as diarrhea and nausea, were observed. One patient, who was allergic to a drug-lymphocyte product, had liver impairment. None of these side effects appeared to be dose-dependent. There was no immune result implemented.
• Torkelson et al. (Torkelson et al., 2012) used powdered C. *versicolor* mycelium in a phase I two-center dose-escalation clinical analysis to determine the extremely tolerable dosage when given regularly in divided dosages for 6 weeks to women who had breast cancer who had completed standardized chemotherapy and radiotherapy. Nine people were divided into three groups and given 3, 6, or 9 g of medicinal mushroom powder (500 mg of lyophilized mycelial capsule/powder). Each of these three dosages was neatly authorized. Out of which 9 antagonistic events were described, 7 of them were mild, and 1 of which was moderate, and one was severe (anxiety, which was most likely unrelated to the treatment). Enhanced lymphocyte numbers at a 6 and 9 g/day, enhanced NK cell functional activity at the 6 g/day, and a dosage-associated rise in CD19^+^ B cells and CD8^+^ T cells, but not CD16 + 56 + NK cells or CD4^+^ T cells, were all beneficial impacts on the immune system. Although this trial demonstrated the safety and efficacy of C. *Versicolor* at a regular daily dosage of 9 g, it didn’t assess the safety and acceptability of higher doses, i.e. the extreme dose tolerated (MDT), and the sampling extent was limited.

### 8.2 Clinical Studies on Medicinal Mushrooms and Diabetes, Hyperglycemia, Hyperlipidemia, and Cardiovascular Disorders

• In a random, double-blinded, placebo-controlled experiment including 72 people who has type 2 diabetes who were medicated with prescribed gliclazide and metformin for more than 6 months, the efficacy of A. blazei Murrill in diabetes control was proven (Hsu et al., 2007). The Agaricus blazei mushroom (ABM) extraction was given at a dosage of 1500 mg/d for 12 weeks, and the homeostasis model valuation for insulin resistance (HOMA-IR) were used to examine results. The treatment of the ABM extract was related to a noteworthy reduction in the insulin resistance, which was most likely owing to a rise in the plasma adiponectin concentration, which improved in the ABM group but dropped in the placebo group.
• Kathun et al. (Dicks and Ellinger, 2020)studied the ability of Pleurotus ostreatus to lower cholesterol blood glucose, and the triglycerides in diabetic individuals, as well as possible renal and hepatic damage. Clinical research was directed with 89 participants who consumed 50 g of boiled cooked mushrooms three times regularly for the 24 days, alternate 7 days of cooked mushroom diet with no mushrooms for 7 days, and evaluated several limits at the beginning and end of each 7 days. Patients experienced substantial reductions in systolic and diastolic blood pressure, total cholesterol (TC), triglycerides (TGs), and plasma glucose, but no significant changes in weight or HDL. FPG, DBP, and PG 2 h after breakfast (2 hPG), TGs, and TC, all increased significantly when mushrooms were not consumed, although SBP, HDL, and weight remained the same. The above-mentioned modifications recurred when mushroom consumption was resumed. As a result, this fungus can provide significant benefits to diabetic acquired patients without jeopardizing their liver or kidney function.

### 8.3 Medicinal Mushrooms Used in Neuron Health (Clinical Studies)

• G. lucidum is commonly used in Asian regions for the treatment of diabetes, cancer, and neurasthenia, among other ailments. Tang et al. (Tang et al., 2005) conducted a randomized, double-blinded, placebo-controlled clinical trials having Ganopoly^®^ and found that the mushroom has notably positive effects on the latter. For 8 weeks, neurasthenic patients (132) were randomly assigned to obtain either a placebo or 1800 mg 3 times daily. Patients using G. lucidum had a higher sensation of well-being at the end of treatment, as judged by a Visual Analogue Scale (VAS), as well as a consistent reduction in the Clinical Global Impression (CGI) severity scale.
• Nagano et al. (Nagano et al., 2010)evaluated the resultsof H*. erinaceus* (HE) on depression, sleep quality, menopause, and unexplained diseases in a random, doubleblinded, placebo-controlled experiment because of its effect on the (autonomic) nervous system and brain function. The Kupperman Menopausal Index (KMI), the Center for Epidemiologic Studies Depression Scale (CES-D), the Pittsburgh Sleep Quality Index (PSQI), and the Indefinite Complaints Index were used to conduct the assessments (ICI). For 1 month, thirty women were randomly allotted to eat either four placebo cookies or four HE cookies (0.5 g of powdered carpophore each cookie). The ICI and CES-D scores in the treatment group were notably lower than before the HE intake; in comparison to the placebo group, the ICI words “insensitive” and “palpitation” were remarkably lower, while the phrases “concentration,” “irritating,” and “anxious” tends to be lower. When the authors discovered that the H. erinaceus helps with anxiety, panic, and depression, they led to believe that the mushroom’s mode of action remained different from the NGF-enhancing activity.

### 8.4 Toxicological Profile of various Medicinal Mushrooms

Mushrooms, being the source of secondary metabolites, also have the long history for being used as culinary and medicinal agents. They have been valued for their dietary and medicinal prospective. They have been reported to have cardiovascular, anticancer, anti allergic nti asthmatic and many more pharmacological action. However, the knowledge of their toxicity data and human evaluation dose data is not sufficient (Kuroiwa et al., 2005), (Vander et al., 2011), (Venturella et al., 2021).

❖ *Ganoderma Lingzhi:* Numerous toxicological studies were performed on Reishi mushrooms by a number of researchers. It had been concluded that the 90 days studies on the polysaccharide fraction of the mushrooms provide NOALs (No observed Adverse levels) at a dose of 1200 mg/kg and 2000 mg/kg. Also, this non toxicity was also supported by the literature review on the same.
❖ *Agaricus Subrufescens and Agaricus Blazei:* According to the study performed on *A. Subrufescens and A. Blazei,,* no toxicity was observed at the dose of 2000 mg/kg in either species. However, decrease in locomotor activity was observed in some rats who were receiving the dose of 2000 mg/k of A. Subrufescens. (Moukha et al., 2011).

## 9 Fungal Nanobiotechnology

• In recent years, there has been a boost in the acceptability of nanotechnology in biomedicine, which has resulted in the rapid growth of fungal nanobiotechnology in biomedical sciences. Nanomedicine research has evolved gradually and dramatically, from diagnosis to treatment and medication delivery. Fungal nanobiotechnology has found uses in biologically labeled fluorescent, gene, and drug delivery factors, as well as pathogen detection, tissue engineering, tumor destruction by heating (hyperthermia), MRI contrast enhancement, and phagokinetic studies. With discrete nanoparticles commonly used in nano biomedicine, magnetic nanoparticles are useful for targeted drug delivery and hyperthermia. The availability of various journals and articles on the use of fungal nanoparticles in biomedicine has aided progress significantly over time. Because biosynthesized nanoparticles are such a new subject, researchers are already looking at their potential uses in areas including drug delivery, cancer therapy, gene treatment and DNA analysis, antimicrobial, biological sensors, separation science, and MRI. (Mishra et al., 2011).used the supernatant, live-cell filtrate, and biomass to explore the manufacture of AuNP in the fungus Penicillium brevicompactum, whereas the effects of Ag-NP on cancer cell lines in another study. Silver nanoparticles with diameters ranging from 5 to 40 nm were naturally produced using the fungus Trichoderma viride (Kumar et al., 2007).
• The capacity of nanoparticles to increase the antibacterial action of a variety of antibiotics against Gram-negative and Gram-positive bacteria has also been investigated. Antibacterial activities of kanamycin, ampicillin, erythromycin, and chloramphenicol were enhanced when combined with Ag-NP in contradiction of pathogenic species, with ampicillin having the greatest effect. Combining antibiotics with Ag-NP increased antibacterial action, according to this result. (Duhan et al., 2017). found that extracellularly produced silver nanoparticles of Fusarium oxysporum had an antagonistic impact against *Staphylococcus aureus*, suggesting that this technology could be used in the textile sector in addition to biological applications. Moreover, various researchers have reported on nanotechnology and its influence on pharmaceutical development, as well as metallic nanoparticles and their wide variety of potentials in pharmaceutical applications such as antiparasite, bactericidal, anti-cancer, fungicidal, and so on. (Chopra et al., 2022). published a systematic assessment of the efficacy of these nanoparticles (NPs) against lung cancer using *in vitro* models (2019). The ability to biosynthesize nanoparticles (NPs) from Penicillium species has been regarded as a possible technique for controlling malaria vectors as well as a malaria treatment (Barabadi et al., 2019). (Khatua et al., 2020) used Pongamia pinnata leaf extract as a unique strategy in nanoparticle synthesis to investigate the fungicidal capabilities of emerging gold nanoparticles. The findings revealed a considerable inhibitory capability against plant pathogens, suggesting that it could be used as a plant pathogen antifungal agent. Metallic nanoparticles mediated by mushroom fruiting bodies have been identified with various biomedical uses (Adebayo et al., 2021).

## 10 Perspectives, Trends, Research Gaps, and the Unresolved Problem

• Moreover, mushroom science has advanced significantly in the previous 30 years, embracing both TCM and Chinese herbal medicines, as well as their commercial adaptations. Meanwhile, there are unanswered queries regarding its safety, efficacy, and critical issues that affect the future mushroom medicine development, that could jeopardize its usage in the twenty-first century. We have compiled a list of the most pressing issues confronting MM science’s future progress. (IntJMedMushr.V12.I1.10, 2022).

The development of mushroom carbohydrate polymers such as DSs or functional meals has recently gained attention. When it comes to developing DS and MM goods, there are several things to consider, including their regulation, safety, standardization, efficacy, and mode of action.

• Conversely, medicinal mushrooms/fungi are still being standardized over the world, and there is a lack of understanding of their bioactive effects. For the development and testing of fungus products, there are no internationally recognized standards and methods. Product quality can only be ensured by adhering to proper standards and regulations. Commercially available medicine fungus (mainly mushrooms), preparations of mushrooms will be drastically diverse and vary enormously in composition and affectivity if the quality of medicinal fungus goods is not consistent. It’s uncertain if pharmacological effects are caused by a single substance or by a synergistic action of several ingredients. There is not sufficient statistical data to note which components of fungus eg-fruiting bodies, mycelia powder or its extracts are as effective as hot water, alcoholic, or hydro-alcoholic extracts. The role of lowmolecular-weight compounds for fungus extracts is still unclear.
• The study of cooking medicinal mushrooms/fungus in pure culture requires extra attention. Cultural research is required for scientific activity to be stable and consistent. Although the teleomorph stage is the most important requirement for identifying cultures, fungi do not always form fruiting bodies in pure culture. In mycological literature, vegetative mycelia of fungus in pure culture received much less attention. Many fungal species cannot be reliably noticed without the analysis of vegetative mycelia. (*IntJMedMushr.V12.I1.10*, n. d.).
• The utilization of high-molecular-weight molecules has impeded the growth of true immunomodulating and anticancer medicines from MM, polysaccharides (e.g., Lentinan, Schizophyllan, and Krestin). All medicinal fungi (medicinal mushroom) medicines are made up of polysaccharides with molecular weights ranging from 100,000 to 0.5 million Da.

Because these substances are not produced, they can only be extracted from fruit bodies, cultured broth, or cultured mycelium. Such a strategy necessitates high market prices. Medicinal mushrooms produced low-weight molecular compounds, such as low-molecularweight, secondary metabolites that target processes including angiogenesis, metastasis, apoptosis, cell cycle regulation, and signal transduction cascades, which should be the focus of science today. (Zaidman et al., 2005).

• The success of -glucans and another type of mushroom carbohydrate polymer use depends on an ongoing study into the structural-activity relation of mushrooms carbohydrate polymers, especially in terms of receptor-mediated and molecular conformation processes (Chen and Seviour, 2007). Water solubility, molecule size and molecular weight, structure, and molecular processes of -glucan action are all clarified to account for the fact that not all glucans in an MM have medisave. The function of molecular weights in the medicinal activity of glucans is unknown. There are still problems with the affectivity of highmolecular-weight glucans versus low-molecular-weight glucans. The most effective scleroglucan provisions are those with a high molecular weight. (Bashir & Choi, 2017). Only low-molecular-weight Lentinan, for example, has a stronger anticancer action. (Kim et al., 2022) (Mantovani et al., 2008).The varied reactivity of -glucans in each distinct must be taken into account.

For the properties of -glucans, still, it is not known which primary elements influence -glucan solubility and pharmacological activity: The molecular weight, side-chain length, number of side chains on the main chain, ratios of (1,4), (1,6), and (1,3)-linkages, and acid ionization must all be taken into account. (Wasser, 2002; Cavalu et al., 2002), Insoluble -glucans appear to be less effective immunostimulants than soluble glucans. The details for this are a little hazy. The precise mechanism of orally administered glucan intestinal absorption (passage of glucans in the gap junction in the intestinal epithelial membrane; nonspecific intestinal absorption; absorption in the intestinal M cells; absorption after binding with Toll-like receptor proteins on the intestinal lumen; and dendritic cell probing) remains unknown. (Cavalu et al., 2002; Miller et al., 2007), After intake, it’s likely that insoluble -glucans are reduced into smaller bioactive oligomers. (Lehmann, 2022). Plant -glucans, (Tiwari and Cummins, 2009; Tada et al., 2008),yeast -glucans (Vetvicka and Vetvickova, 2009; Liu et al., 2009), and -glucans from MMs must all be distinguished. (M. Zhang et al., 2007; Chen & Seviour, 2007). What are the structural, solubility, and biological activity differences? Cereal -glucan, for example, is primarily made up of 1,3 and 1,4-linkages, rather than 1,6linkages. Plant -glucans are also linear rather than branching. Plant-glucan molecular weights are often lower than MM-glucan molecular weights. In the case of plant -glucans, biological activity has not been well investigated. Various MM glucans are water-insoluble, whereas yeast-glucans are partially water-soluble.

## 11 Conclusion

In complement to its vital nutritional significance, numerous mushroom species of medicinal value have been identified as sources of bioactive chemicals. Incorporating entire mushrooms into one’s diet could be beneficial as a nutritional supplement. Many research has proved that the mushrooms encompass components that has exceptional possessions for preventing or treating various ailments. The mushrooms with medicinal values contain a number of primary and secondary bioactive metabolites owing to which, the mushrooms posses various therapeutic activities such as anticancer, antiviral and anti-hypertensive actions. the same was envisaged from the clinical data as well. Also, the acute toxicity studies were performed on various mushrooms and it was evaluated that the most of the therapeutically active mushrooms are safe at a dose of 2000 mg/kg with mild side effects.

Only a few mushrooms’ pharmaceutical properties have been scrutinized in recent decades, so there is still a lot to learn. Furthermore, high-quality, double-blind, randomized, long-term, placebo-controlled human clinical investigations with big sample numbers and adequate power are required, as well as current statistical and bioinformatics tools. More research is necessary to determine which mushroom extracts are most beneficial in treating various cancers.

## References

[B1] Abdel-Azeem A. M. Abdel-Azeem M. A. Khalil W. F. (2019). “Endophytic Fungi as a New Source of Antirheumatoid Metabolites,” in Bioactive Food as Dietary Interventions for Arthritis and Related Inflammatory Diseases (Amsterdam, Netherlands: Elsevier), 355–384. 10.1016/b978-0-12-813820-5.00021-0

[B2] Abidin M. H. Z. Abdullah N. Abidin N. Z. (2017). Therapeutic Properties ofPleurotusspecies (Oyster Mushrooms) for Atherosclerosis: A Review. Int. J. Food Prop. 20 (6), 1251–1261. 10.1080/10942912.2016.1210162

[B3] Adams L. S. Phung S. Wu S. Ki X. Chen L. (2008). White Button Mushroom (*Agaricus Bisporus*) Exhibits Antiproliferative and Proapoptotic Properties and Inhibits Prostate Tumor Growth in Athymic Mice. Nutr. Cancer 60 (6), 744–756. 10.1080/01635580802192866 19005974

[B4] Adebayo E. A. Azeez M. A. Alao M. B. Oke A. M. Aina D. A. (2021)., 7. Elsevier, e08480. 10.1016/j.heliyon.2021.e08480 Fungi as Veritable Tool in Current Advances in Nanobiotechnology Heliyon 11 34901509 PMC8640478

[B5] Ahn W.-S. Kim D.-J. Chae G.-T. Lee J.-M. Bae S.-M. Sin J.-I. (2004). Natural Killer Cell Activity and Quality of Life Were Improved by Consumption of a Mushroom Extract, Agaricus Blazei Murill Kyowa, in Gynecological Cancer Patients Undergoing Chemotherapy. Int. J. Gynecol. Cancer 14, 589–594. 10.1111/j.1048-891X.2004.14403.x 15304151

[B6] Anil Kumar S. A. Abyaneh M. K. Gosavi S. W. Kulkarni S. K. Pasricha R. Ahmad A. (2007). Nitrate Reductase-Mediated Synthesis of Silver Nanoparticles from AgNO3. Biotechnol. Lett. 29 (3), 439–445. 10.1007/s10529-006-9256-7 17237973

[B7] Awadasseid A. Hou J. Gamallat Y. Xueqi S. Eugene K. D. Musa Hago A. M. (2017). Purification, Characterization, and Antitumor Activity of a Novel Glucan from the Fruiting Bodies of Coriolus Versicolor. PloS ONE 12 (2), e0171270. 10.1371/journal.pone.0171270 28178285 PMC5298263

[B8] Ayeka P. A. (20182018). Potential of Mushroom Compounds as Immunomodulators in Cancer Immunotherapy: A Review. Evidence-Based Complementary Altern. Med. 2018, 1–9. 10.1155/2018/7271509 PMC5937616 29849725

[B9] Badalyan S. M. Barkhudaryan A. Rapior S. (2019). “Recent Progress in Research on the Pharmacological Potential of Mushrooms and Prospects for Their Clinical Application,” in Medicinal Mushrooms (Singapore: Springer), 1–70. 10.1007/978-981-136382-5_110.1007/978-981-13-6382-5_1

[B10] Balandaykin M. E. Zmitrovich I. v. (2015). Review on Chaga Medicinal Mushroom, Inonotus Obliquus (Higher Basidiomycetes): Realm of Medicinal Applications and Approaches on Estimating its Resource Potential. Int. J. Med. Mushrooms 17 (2), 95–104. 10.1615/IntJMedMushrooms.v17.i2.10 25746615

[B11] Barabadi H. Tajani B. Moradi M. Damavandi Kamali K. Meena R. Honary S. (2019). Penicillium Family as Emerging Nanofactory for Biosynthesis of Green Nanomaterials: A Journey into the World of Microorganisms. J. Clust. Sci. 30 (4), 843–856. 10.1007/s10876-019-01554-3

[B12] Bashir K. M. I. Choi J. S. (2017). Clinical and Physiological Perspectives of β-Glucans: The Past, Present, and Future. Int. J. Mol. Sci. 18 (9). 10.3390/ijms18091906 PMC5618555 28872611

[B13] Bhattacharya T. Soares G. A. B. e. Chopra H. Rahman M. M. Hasan Z. Swain S. S. (2022). Applications of Phyto-Nanotechnology for the Treatment of Neurodegenerative Disorders. Mater. (Basel) 15, 804. 10.3390/ma15030804 PMC8837051 35160749

[B14] Bhunia S. K. Dey B. Maity K. K. Patra S. Mandal S. Maiti S. (2011). Isolation and Characterization of an Immunoenhancing Glucan from Alkaline Extract of an Edible Mushroom, Lentinus Squarrosulus (Mont.) Singer. Carbohydr. Res. 346 (13), 2039–2044. 10.1016/j.carres.2011.05.029 21722881

[B15] Bimczok D. Wrenger J. Schirrmann T. Rothkötter H. J. Wray V. Rau U. (2009). Short Chain Regioselectively Hydrolyzed Scleroglucans Induce Maturation of Porcine Dendritic Cells. Appl. Microbiol. Biotechnol. 82 (2), 321–331. 10.1007/s00253008-1813-710.1007/s00253-008-1813-7 19107473

[B16] Blumfield M. Abbott K. Duve E. Cassettari T. Marshall S. Fayet-Moore F. (2020). Journal of Nutritional Biochemistry, 84, 108453. 10.1016/j.jnutbio.2020.108453 Examining the Health Effects and Bioactive Components in Agaricus Bisporus Mushrooms: a Scoping Review J. Nutr. Biochem. 32653808

[B17] Cavalu S. Antoniac I. V. Mohan A. Bodog F. Doicin C. Mates I. (2020). Nanoparticles and Nanostructured Surface Fabrication for Innovative Cranial and Maxillofacial Surgery. Mater. (Basel) 13, 5391. 10.3390/ma13235391 PMC7731022 33260938

[B18] Cavalu S. Bisboaca S. Mates I. M. Pasca P. M. Laslo V. Costea T. (2018). Novel Formulation Based on Chitosan-Arabic Gum Nanoparticles Entrapping Propolis Extract Production, Physico-Chemical and Structural Characterization. Rev. Chim. 69 (12), 3756–3760.

[B86] Cavalu S. Damian G. Dansoreanu M. (2002). EPR Study of Non-Covalent Spin Labeled Serum Albumin and Hemoglobin. Biophys. Chem. 99 (2), 181–188. 10.1016/S0301-4622(02)00182-5 12377368

[B19] Chen J. Seviour R. (2007). Medicinal Importance of Fungal β-(1→3), (1→6)-glucans. Mycol. Res. 111 (6), 635–652. 10.1016/j.mycres.2007.02.011 17590323

[B20] Choi B. S. Sapkota K. Choi J. H. Shin C. H. Kim S. Kim S. J. (2013). Herinase: A Novel Bi-functional Fibrinolytic Protease from the Monkey Head Mushroom, Hericium Erinaceum. Appl. Biochem. Biotechnol. 170 (3), 609–622. 10.1007/s12010013-0206-210.1007/s12010-013-0206-2 23564433

[B21] Choi H. S. Cho H. Y. Yang H. C. Ra K. S. Suh H. J. (2001). Angiotensin I-Converting Enzyme Inhibitor from Grifola 24 Rondose. Food Res. Int. 34 (2-3), 177–182. 10.1016/s0963-9969(00)00149-6

[B22] Chopra H. Bibi S. Singh I. Hasan M. M. Khan M. S. Yousafi Q. (2022). Green Metallic Nanoparticles: Biosynthesis to Applications. Front. Bioeng. Biotechnol. 10, 874742. 10.3389/fbioe.2022.874742 35464722 PMC9019488

[B23] Corrêa R. C. G. Brugnari T. Bracht A. Peralta R. M. Ferreira I. C. (2016). Biotechnological, Nutritional and Therapeutic Uses of Pleurotus spp.(Oyster Mushroom) Related with its Chemical Composition: A Review on the Past Decade Findings. Trends Food Sci. Technol. 50, 103–117.

[B24] Cui W. Aouidate A. Wang S. Yu Q. Li Y. Yuan S. (2020). Discovering Anti-cancer Drugs via Computational Methods. Front. Pharmacol. 11, 733. 10.3389/fphar.2020.00733 32508653 PMC7251168

[B25] Cunha Zied D. Pardo-Giménez A. (2017). Edible and Medicinal Mushrooms: Technology and Applications. Funct. Foods Nutraceuticals 4 (12), 3756–3760.

[B26] da Silva de Souza C. S. Correa G. Goncalves D. A. Soares A. Bracht A. Peralta M. (2017). Agaricus Blazei Bioactive Compounds and Their Effects on Human Health: Benefits and Controversies. Curr. Pharm. Des. 23 (19), 2807–2834. 10.2174/1381612823666170119093719 28103773

[B27] Deshmukh S. K. Prakash V. Ranjan N. (2018). Frontiers in Microbiology, 8. 10.3389/fmicb.2017.02536 Marine Fungi: A Source of Potential Anticancer Compounds Front. Microbiol. JAN PMC5760561 29354097

[B28] Dicks L. Ellinger S. (2020). Effect of the Intake of Oyster Mushrooms (Pleurotus Ostreatus) on Cardiometabolic Parameters-A Systematic Review of Clinical Trials. Nutrients 12 (4). 10.3390/nu12041134 PMC7230384 32316680

[B29] Duhan J. S. Kumar R. Kumar N. Kaur P. Nehra K. Duhan S. (2017). Nanotechnology: The New Perspective in Precision Agriculture. Biotechnol. Rep. (Amst) 15, 11–23. 10.1016/j.btre.2017.03.002 28603692 PMC5454086

[B30] El Enshasy H. A. Hatti-Kaul R. (2013). Mushroom Immunomodulators: Unique Molecules with Unlimited Applications. Trends Biotechnol. 31, 668–677. 10.1016/j.tibtech.2013.09.003 24125745

[B31] Ely S. W. Berne R. M. (1992). Protective Effects of Adenosine in Myocardial Ischemia. Circulation 85 (3), 893–904. 10.1161/01.cir.85.3.893 1537125

[B32] Firenzuoli F. Gori L. Lombardo G. (2008). The Medicinal Mushroom Agaricus Blazei Murrill: Review of Literature and Pharmaco-Toxicological Problems. Evid. Based Complement. Altern. Med. 5 (1), 3–15. 10.1093/ecam/nem007 PMC2249742 18317543

[B33] Fortes R. C. Novaes R. C. Recôva V. L. Melo l. (2009). Immunological, Hematological, and Glycemia Effects of Dietary Supplementation with Agaricus Sylvaticus on Patients' Colorectal Cancer. Exp. Biol. Med. (Maywood) 234 (1), 53–62. 10.3181/0806-RM-193 18997106

[B34] FUNGI USED AS FOOD History of mushroom use (2020). FUNGI USED AS FOOD History of Mushroom Use.

[B35] Ganeshpurkar A. Rai G. Jain A. (2010). Medicinal Mushrooms: Towards a New Horizon. Pharmacogn. Rev. 4 (8), 127–135. 10.4103/09737847.7090410.4103/0973-7847.70904 22228952 PMC3249912

[B36] Gill B. S. Sharma P. Kumar R. Kumar S. (2016). Misconstrued Versatility of Ganoderma Lucidum: a Key Player in Multi-Targeted Cellular Signaling. Tumour Biol. 37, 2789–2804. 10.1007/s13277-015-4709-z 26715282

[B37] Goodridge H. S. Wolf A. J. Underhill D. M. (2009). Beta-glucan Recognition by the Innate Immune System. Immunol. Rev. 230 (1), 38–50. 10.1111/j.1600-065X.2009.00793.x 19594628 PMC6618291

[B38] Guggenheim A. G. Wright K. M. Zwickey H. L. (2014). Immune Modulation from Five Major Mushrooms: Application to Integrative OncologyIntegrative Medicine. Integr. Med. (Encinitas) 13 (1), 32–44. PMC4684115 26770080

[B39] Hameed A. Hussain S. A. Yang J. Ijaz M. U. Liu Q. Suleria H. A. R. (2017). Antioxidants Potential of the Filamentous Fungi (Mucor Circinelloides). Nutrients 9 (10). 10.3390/nu9101101 PMC5691717 28991177

[B40] He X. Wang X. Fang J. Chang Y. Ning N. Guo H. (2017). Structures, Biological Activities, and Industrial Applications of the Polysaccharides from Hericium erinaceus (Lion's Mane) Mushroom: A Review. Int. J. Biol. Macromol. 97, 228–237. 10.1016/j.ijbiomac.2017.01.040 28087447

[B41] Hetland G. Johnson E. Bernardshaw S. v. Grinde B. (2021). Can Medicinal Mushrooms Have Prophylactic or Therapeutic Effect against COVID-19 and its Pneumonic Superinfection and Complicating Inflammation? Scand. J. Immunol. 93 (1), e12937. 10.1111/sji.12937 32657436 PMC7404338

[B42] Hosseini M. Salam A. Makhlouf H. (2016). Industrial Applications for Intelligent Polymers and Coatings. Berlin, Germany: Spinger.

[B43] Hsu C. H. Liao Y. L. Lin S. C. Hwang K. C. Chou P. (2007). The Mushroom Agaricus Blazei Murill in Combination with Metformin and Gliclazide Improves Insulin Resistance in Type 2 Diabetes: A Randomized, Double-Blinded, and Placebo-Controlled Clinical Trial. J. Altern. Complement. Med. 13 (1), 97–102. 10.1089/acm.2006.6054 17309383

[B44] Inácio F. D. Ferreira R. O. Araujo C. A. V. D. Brugnari T. Castoldi R. Peralta R. M. (2015). BioMed Research International, 2015, 1–10. 10.1155/2015/290161 Proteases of Wood Rot Fungi with Emphasis on the GenusPleurotus BioMed Res. Int. PMC4477095 26180792

[B45] IntJMedMushr.v12.i1.10 (2022). IntJMedMushr.v12.i1.10.

[B46] Jeong Y.-T. Yang B.-K. Jeong S.-C. Kim S.-M. Song C.-H. (2008). Ganoderma Applanatum: A Promising Mushroom for Antitumor and Immunomodulating Activity. Phytother. Res. 22, 614–619. 10.1002/ptr10.1002/ptr.2294 18398900

[B47] Kang J. H. Jang J. E. Mishra S. K. Lee H. J. Nho C. W. Shin D. (2015). Ergosterol Peroxide from Chaga Mushroom (Inonotus Obliquus) Exhibits Anti-cancer Activity by Down-Regulation of the β-catenin Pathway in Colorectal Cancer. J. Ethnopharmacol. 173, 303–312. 10.1016/j.jep.2015.07.030 26210065

[B48] Kawagishi H. Nomura A. Mizuno T. Kimura A. Chiba S. (1990). “Isolation and Characterization of a Lectin from Grifola Frondosa26rondose Fruiting Bodies,” in Biochimica et Biophysica Acta. 10.1016/0304-4165(90)90045-x 2364082

[B49] Khan M. A. Tania M. (2012). Nutritional and Medicinal Importance ofPleurotusMushrooms: An Overview. Food Rev. Int. 28 (3), 313–329. 10.1080/87559129.2011.637267

[B50] Khatua A. Priyadarshini E. Rajamani P. Patel A. Kumar J. Naik A. (2020). Phytosynthesis, Characterization and Fungicidal Potential of Emerging Gold Nanoparticles Using Pongamia Pinnata Leave Extract: A Novel Approach in Nanoparticle Synthesis. J. Clust. Sci. 31 (1), 125–131. 10.1007/s10876-019-01624-6

[B51] Khatua S. Dutta A. K. Chandra S. Paloi S. Das K. Acharya K. (2017). Introducing a Novel Mushroom from Mycophagy Community with Emphasis on Biomedical Potency. Plos One 12 (5), e0178050–25. 10.1371/journal.pone.0178050 28552988 PMC5446119

[B52] Khatun S. Islam A. Cakilcioglu U. Chatterjee N. C. (2012). Research on Mushroom as a Potential Source of Nutraceuticals: A Review on Indian PerspectiveReview Article American Journal of Experimental Agriculture. J. Exp. Agric. Int. 2, 1. 10.9734/ajea/2012/492

[B53] Kim S. P. Kang M. Y. Kim J. H. Nam S. H. Friedman M. (2011). Composition and Mechanism of Antitumor Effects of Hericium erinaceus Mushroom Extracts in Tumor-Bearing Mice. J. Agric. Food Chem. 59 (18), 9861–9869. 10.1021/jf201944n 21846141

[B54] Kim S. Y. Song H. J. Lee Y. Y. (2022). SEARCH STRATEGY. Available at:http://www.

[B55] Kück U. Bloemendal S. Teichert I. (2014). Putting Fungi to Work: Harvesting a Cornucopia of Drugs, Toxins, and Antibiotics. PLoS Pathog. 10 (3), e1003950. 10.1371/journal.ppat.1003950 24626260 PMC3953401

[B56] Kumar K. Mehra R. Guiné R. P. F. Lima M. J. Kumar N. Kaushik R. (2021). Edible Mushrooms: A Comprehensive Review on Bioactive Compounds with Health Benefits and Processing Aspects. Foods 10 (16), 2996. 10.3390/foods10122996 34945547 PMC8700757

[B57] Kumaran S. Palani P. Nishanthi R. Kaviyarasan V. (2011). Studies on Screening, Isolation and Purification of a Fibrinolytic Protease from an Isolate (VK12) of Ganoderma Lucidum and Evaluation of its Antithrombotic Activity. Med. Mycol. J. 52, 153–162. 10.3314/jjmm.52.153 21788727

[B58] Kurowa Y. Nishikawa A. Kanki K. Kitamura Y. Umemura T. (2005). Lack of Subchronic Toxicity of an Aqueous Extract of Agaricus Blezeii Murill in F344 Rats. Food Chem. Toxicol. 43 (7), 1047–1053. 15833380 10.1016/j.fct.2005.02.007

[B59] Lau C. C. Abdullah N. Shuib A. S. Aminudin N. (2014). Novel Angiotensin I-Converting Enzyme Inhibitory Peptides Derived from Edible Mushroom Agaricus Bisporus (J.E. Lange) Imbach Identified by LC-MS/MS. Food Chem. 148, 396–401. 10.1016/j.foodchem.2013.10.053 24262574

[B60] Lee K.-H. Morris-Natschke S. L. Yang X. Huang R. Zhou T. Wu S.-F. (2011). Recent Progress of Research on Medicinal Mushrooms, Foods, and Other Herbal Products Used in Traditional Chinese Medicine. J. Traditional Complementary Med. 1 (1). PMC3942920 24716120

[B61] Lehmann (2022). Patent Number: Date of Patent: United States Patent. US patent no. US10278935B2. (Accessed May 07, 2019).

[B62] Lin C. H. Hsiao Y. M. Ou C. C. Lin Y. W. Chiu Y. L. Lue K. H. (2010). GMI, a Ganoderma Immunomodulatory Protein, Down-Regulates Tumor Necrosis Factor α-induced Expression of Matrix Metalloproteinase 9 via NF-Κb Pathway in Human Alveolar Epithelial A549 Cells. J. Agric. Food Chem. 58 (22), 12014–12021. 10.1021/jf103068w 21028821

[B63] Liu J. Gunn L. Hansen R. Yan J. (2009). Combined Yeast-Derived Beta-Glucan with Anti-tumor Monoclonal Antibody for Cancer Immunotherapy. Exp. Mol. Pathol. 86 (3), 208–214. 10.1016/j.yexmp.2009.01.006 19454271 PMC2685877

[B64] Liu Q. Wang H. Ng T. B. (2006). First Report of a Xylose-specific Lectin with Potent Hemagglutinating, Antiproliferative and Anti-mitogenic Activities from a Wild Ascomycete Mushroom. Biochim. Biophys. Acta 1760 (12), 1914–1919. 10.1016/j.bbagen.2006.07.010 16952421

[B65] Loud J. T. Murphy J. (2017). Cancer Screening and Early Detection in the 21st Century. Semin. Oncol. Nurs., 33(2), 121–128. 10.1016/j.soncn.2017.02.002 28343835 PMC5467686

[B66] Lu C.-L. Chen S.-N. (2012). Fibrinolytic Enzymes from Medicinal Mushrooms. Available at: www.intechopen.com. 10.5772/38221

[B67] Lucius K. (2020). Medicinal Mushrooms: Current Use in Clinical Practice. Altern. Complementary Ther. 26 (3), 119–126. 10.1089/act.2020.29275.kha

[B68] Lull C. Wichers H. J. Savelkoul H. F. J. (2005). Antiinflammatory and Immunomodulating Properties of Fungal Metabolites. Mediat. Inflamm. 2005 (2), 63–80. 10.1155/MI.2005.63 PMC1160565 16030389

[B69] Ma G. Kimatu B. M. Zhao L. Yang W. Pei F. Hu Q. (2017). *In Vivo* fermentation of a Pleurotus Eryngii Polysaccharide and its Effects on Fecal Microbiota Composition and Immune Response. Food Funct. 8 (5), 1810–1821. 10.1039/c7fo00341b 28513745

[B70] Ma K. Bao L. Han J. Jin T. Yang X. Zhao F. (2014). New Benzoate Derivatives and Hirsutane Type Sesquiterpenoids with Antimicrobial Activity and Cytotoxicity from the Solid-State Fermented Rice by the Medicinal Mushroom Stereum Hirsutum. Food Chem. 143, 239–245. 10.1016/j.foodchem.2013.07.124 24054236

[B71] Ma Z. Wang J. Zhang L. Zhang Y. Ding K. (2010). Evaluation of Water Soluble β-d-glucan from Auricularia Auricular-Judae as Potential Anti-tumor Agent. Carbohydr. Polym. 80 (3), 977–983. 10.1016/j.carbpol.2010.01.015

[B72] Mantovani M. S. Bellini M. F. Angeli J. P. F. Oliveira R. J. Silva A. F. Ribeiro L. R. (2008). Beta-Glucans in Promoting Health: Prevention against Mutation and Cancer. Mutat. Res. 658 (3), 154–161. 10.1016/j.mrrev.2007.07.002 17827055

[B73] Markham M. J. Wachter K. Agarwal N. Bertagnolli M. M. Chang M. Dale W. (2020). Clinical Cancer Advances 2020: Annual Report on Progress against Cancer from the American Society of Clinical Oncology. J. Clin. Oncol. 38, 1081. 10.1200/JCO.1910.1200/JCO.19.03141 32013670

[B74] Melappa K. K. Murugan C. C. Poojari C. Ryavalad R. Y. Lakshmikan P. R. Satwadi R. R. (2017). Anti-diabetic Activity of Endophytic Fungi, Penicillium Species of Tabebuia Argentea; In Silico and Experimental Analysis. Res. J. Phytochemistry 11 (2), 90–110. 10.3923/rjphyto.2017.90.110

[B75] Miller H. Zhang J. Kuolee R. Patel G. B. Chen W. (2007). Intestinal M Cells: The Fallible Sentinels? World J. Gastroenterol. 13 (10), 1477–1486. 10.3748/wjg.v13.i10.1477 17461437 PMC1876659

[B76] Milovanovic N. Stanojkovic I., P. Stajic T., M. Brceskic M., D. Knezevic I., Z. Cilerdzic A., Lj. (2015). Effect of Selenium Enrichment of Lenzites Betulinus and Trametes Hirsuta Mycelia on Antioxidant, Antifungal and Cytostatics Potential. Curr. Pharm. Biotechnol. 16 (10), 920–926. 10.2174/1389201016666150618152531 26087835

[B77] Mishra A. Tripathy S. K. Wahab R. Jeong S. H. Hwang I. Yang Y. B. (2011). Microbial Synthesis of Gold Nanoparticles Using the Fungus Penicillium brevicompactum and Their Cytotoxic Effects against Mouse Mayo Blast Cancer C 2 C 12 Cells. Appl. Microbiol. Biotechnol. 92 (3), 617–630. 10.1007/s00253-011-3556-0 21894479

[B78] Mizuno K. Nishitani M. Nishitani Y. (2013). Immunomodulating Compounds in Basidiomycetes. J. Clin. Biochem. Nutr. 52 (3), 202–207. 10.3164/jcbn.133310.3164/jcbn.13-3 23704809 PMC3652302

[B79] Money N. P. (2016). Are Mushrooms Medicinal? Fungal Biol. 120 (4), 449–453. 10.1016/j.funbio.2016.01.006 27020147

[B80] Moradali M. F. Mostafavi H. Ghods S. Hedjaroude G. A. (2007). Immunomodulating and Anticancer Agents in the Realm of Macromycetes Fungi (Macrofungi). Int. Immunopharmacol. 7 (6), 701–724. 10.1016/j.intimp.2007.01.008 17466905

[B81] Moukha S. Ferandon C. Mobio T. Creppy E. E. (2011). “Safety Evaluation of Agaricus Subrufeschens Varities and Their Products of Therapeutic Interest or for Disease Prevention,” in Proceedings of 7th international conference on mushroom biology and mushrooms products, 285–296.

[B82] Mustafa F. Chopra H. Baig A. A. Avula S. K. Kumari T. K. (2022). Edible Mushrooms as Novel Therapeutics: Effect on Lipid Level, Obesity, and BMI. J. Fungi 8 (2), 211. 10.3390/jof8020211 PMC8880354 35205965

[B83] Nagano M. Shimizu K. Kondo R. Hayashi C. Sato D. Kitagawa K. (2010). Reduction of Depression and Anxiety by 4 Weeks Hericium erinaceus Intake. Biomed. Res. 31 (4). 10.2220/biomedres.31.231 20834180

[B84] Ohno S. Sumiyoshi Y. Hashine K. Shirato A. Kyo S. Inoue M. (20112011). Phase I Clinical Study of the Dietary Supplement, Agaricus Blazei Murill, in Cancer Patients in Remission. Evidence-Based Complementary Altern. Med. 10.1155/2011/192381 PMC3092499 21584278

[B85] Osińska-Jaroszuk M. Jaszek M. Mizerska-Dudka M. Błachowicz A. Rejczak T. P. Janusz G. (20142014). Exopolysaccharide from Ganoderma Applanatum as a Promising Bioactive Compound with Cytostatic and Antibacterial Properties. BioMed Res. Int. 10.1155/2014/743812 PMC4120920 25114920

[B87] Panda S. K. Sahoo G. Swain S. S. Luyten W. (2022). Anticancer Activities of Mushrooms: A Neglected Source for Drug Discovery. Pharmaceuticals 15 (2), 176. 10.3390/ph15020176 35215289 PMC8876642

[B88] Patel S. Goyal A. (2012). Recent Developments in Mushrooms as Anti-cancer Therapeutics: a Review. 3 Biotech. 2, 1–15. 10.1007/s13205-011-0036-2 PMC3339609 22582152

[B89] Phan C. W. Wang J. K. Cheah S. C. Naidu M. David P. Sabaratnam V. (2018). A Review on the Nucleic Acid Constituents in Mushrooms: Nucleobases, Nucleosides and Nucleotides. Crit. Rev. Biotechnol. 38 (5), 762–777. 10.1080/07388551.2017.1399102 29124970

[B90] Plácido A. I. Roque F. Morgado M. (2022). The Promising Role of Mushrooms as a Therapeutic Adjuvant of Conventional Cancer Therapies. Biologics 2 (1), 58–68. 10.3390/biologics2010005

[B91] Previtali E. Bucciarelli P. Passamonti S. M. Martinelli I. (2011). Risk Factors for Venous and Arterial Thrombosis. Blood Transfus. 9 (2), 120–138. 10.2450/2010.0066-10 21084000 PMC3096855

[B92] Rahman M. A. Abdullah N. Aminudin N. (2016). Interpretation of Mushroom as a Common Therapeutic Agent for Alzheimer’s Disease and Cardiovascular Diseases. Crit. Rev. Biotechnol. 36 (6), 1131–1142. 10.3109/07388551.2015.1100585 26514091

[B93] Rahman M. M. Islam Md. R. Shohag S. Ahasan Md. T. Sarkar N. Khan H. (2022b). Microbiome in Cancer: Role in Carcinogenesis and Impact in Therapeutic Strategies. Biomed. Pharmacother. 149, 112898. 10.1016/j.biopha.2022.112898 35381448

[B94] Rahman M. M. Islam M. R. Shohag S. Hossain M. E. Rahaman M. S. Islam F. (2022a). The Multifunctional Role of Herbal Products in the Management of Diabetes and Obesity: A Comprehensive Review. Molecules 27 (5). 10.3390/molecules27051713 PMC8911649 35268815

[B95] Raja H. A. Miller A. N. Pearce C. J. Oberlies N. H. (2017). Fungal Identification Using Molecular Tools: A Primer for the Natural Products Research Community. J. Nat. Prod. 80 (3), 756–770. 10.1021/acs.jnatprod.6b01085 28199101 PMC5368684

[B96] Seleen A. W. Chen J. (2007). Potential Benefits of Ling Zhi or Reishi Mushroom *Ganoderma Lucidum* (W. Curt.: Fr.) P. Karst. (Aphyllophoromycetideae) to Breast Cancer Patients. Int. J. Med. Mushr 9 (1), 29–38. 10.1615/IntJMedMushr.v9.i1.40

[B97] Shahzad F. Anderson D. Najafzadeh M. (2020). The Antiviral, Anti-inflammatory Effects of Natural Medicinal Herbs and Mushrooms and SARS-CoV-2 Infection. Nutrients 12 (9), 1–13. 10.3390/nu12092573 PMC7551890 32854262

[B98] Shashidhar M. G. Giridhar P. Udaya Sankar K. Manohar B. (2013). Bioactive Principles from Cordyceps Sinensis: A Potent Food Supplement - A Review. J. Funct. Foods 5 (3), 1013–1030. 10.1016/j.jff.2013.04.018 32288795 PMC7104994

[B99] Shibu M. A. Agrawal D. C. Huang C. Y. (2017). “Mushrooms: a Pandora Box of Cardioprotective Phytochemicals,” in Medicinal Plants and Fungi: Recent Advances in Research and Development (Singapore: Springer), 337–362. 10.1007/978-981-10-5978-0_11

[B100] Sindhu R. K. Najda A. Kaur P. Shah M. Singh H. Kaur P. (2021). Potentiality of Nanoenzymes for Cancer Treatment and Other Diseases: Current Status and Future Challenges. Materials 14 (20). 10.3390/ma14205965 PMC8539628 34683560

[B101] Smith R. Sullivan R. (2002). Medicinal Mushrooms: Their Therapeutic Properties and Current Medical Usage with Special Emphasis on Cancer Treatments. San Francisco: Academia.

[B102] Sohretoglu D. Huang S. (2018). Ganoderma Lucidum Polysaccharides as an Anti-cancer Agent. Anti-Cancer Agents Med. Chem. 18 (5), 667–674. 10.2174/1871520617666171113121246 PMC6624854 29141563

[B103] Stajić M. Vukojević J. Ćilerdžić J. (2019). “Mushrooms as Potential Natural Cytostatics,” in Medicinal Mushrooms (Singapore: Springer), 143–168.

[B104] Sugiharto S. Yudiarti T. Isroli I. (2016). Assay of Antioxidant Potential of Two Filamentous Fungi Isolated from the Indonesian Fermented Dried Cassava. Antioxidants 5 (1). 10.3390/antiox5010006 PMC4808755 26848695

[B105] Tada R. Adachi Y. Ishibashi K. I. Tsubaki K. Ohno N. (2008). Binding Capacity of a Barley β-D-glucan to the β-glucan Recognition Molecule Dectin-1. J. Agric. Food Chem. 56 (4), 1442–1450. 10.1021/jf073221y 18205312

[B106] Takaku T. Kimura Y. Okuda H. (2001). Biochemical and Molecular Action of Nutrients Isolation of an Antitumor Compound from Agaricus Blazei Murill and its Mechanism of Action 1. J. Nutr 131. 10.1093/jn/131.5.1409 11340091

[B107] Tang W. Gao Y. Chen G. Gao H. Dai X. Ye J. (2005). A Randomized, Double-Blind and Placebo-Controlled Study of a Ganoderma Lucidum Polysaccharide Extract in Neurasthenia. J. Med. Food 8 (1). 10.1089/jmf.2005.8.53 15857210

[B108] Tiwari U. Cummins E. (2009). Factors Influencing β-glucan Levels and Molecular Weight in Cereal-Based Products. Cereal Chem. 86 (3), 290–301. 10.1094/CCHEM-86-3-0290

[B109] Torkelson C. J. Sweet E. Martzen M. R. Sasagawa M. Wenner C. A. Gay J. (20122012). Phase 1 Clinical Trial of *Trametes versicolor* in Women with Breast Cancer. ISRN Oncol., 1–7. 10.5402/2012/251632 PMC3369477 22701186

[B110] Ulbricht C. Weissner W. Basch E. Giese N. Hammerness P. Rusie-Seamon E. (2009). Maitake Mushroom (Grifola Frondosa): Systematic Review by the Natural Standard Research Collaboration. J. Soc. Integr. Oncol. 7 (2), 66–72. 10.2310/7200.2009.0007 19476741

[B111] Uzma F. Mohan C. D. Hashem A. Konappa N. M. Rangappa S. Kamath P. v. (2018). Frontiers in Pharmacology, 9. 10.3389/fphar.2018.00309 Endophytic Fungi-Alternative Sources of Cytotoxic Compounds: A Review APR PMC5932204 29755344

[B112] Valverde M. E. Hernández-Pérez T. Paredes-López O. (2015). International Journal of Microbiology, 2015. 10.1155/2015/376387 Edible Mushrooms: Improving Human Health and Promoting Quality Life PMC4320875 25685150

[B113] Vander M. Little J. G. Sica J. G. El-Elimat P. S. Raja E. E. Oberlies N. H. (2011). Safety Assessment of Mushrooms in Dietary Supplements by Combining Analytical Data with In Silico Toxicology Evaluation. Food Chem. Toxicol. 103, 133–147. 10.1016/j.fct.2017.03.005 28267567

[B114] Venturella G. Ferraro V. Cirlincione F. Gargano M. L. (2021). Medicinal Mushrooms: BioactiveCompounds, Use, and Clinical Trials. Int. J. Mol. Sci. 22, 634. 10.3390/ijms22020634 PMC7826851 33435246

[B115] Vetvicka V. Vetvickova J. (2009). Effects of Yeast-Derived β-glucans on Blood Cholesterol and Macrophage Functionality Glucans, Blood Cholesterol, and Macrophage Function V. Vetvicka and J. Vetvickova. J. Immunotoxicol. 6 (1), 30–35. 10.1080/15476910802604317 19519160

[B116] Wang S. J. Bao L. Han J. J. Wang Q. X. Yang X. L. Wen H. A. (2013). Pleurospiroketals A-E, Perhydrobenzannulated 5,5-spiroketal Sesquiterpenes from the Edible Mushroom Pleurotus Cornucopiae. J. Nat. Prod. 76 (1), 45–50. 10.1021/np3006524 23294419

[B117] Wasser S. (2002). Medicinal Mushrooms as a Source of Antitumor and Immunomodulating Polysaccharides. Appl. Microbiol. Biotechnol. 60 (3), 258–274. 10.1007/s00253-002-1076-7 12436306

[B118] Wisitrassameewong K. Karunarathna S. C. Thongklang N. Zhao R. Callac P. Moukha S. (2012). Agaricus Subrufescens: A Review. Saudi J. Biol. Sci. 19 (2), 131–146. 10.1016/j.sjbs.2012.01.003 23961172 PMC3730566

[B119] Yahaya N. F. Rahman M. A. Abdullah N. (2014). Therapeutic Potential of Mushrooms in Preventing and Ameliorating Hypertension. Trends Food Sci. Technol. 39 (2), 104–115.

[B120] Yin H. Wang Y. Wang Y. Chen T. Tang H. Wang M. (2010). Purification, Characterization and Immuno-Modulating Properties of Polysaccharides Isolated from Flammulina Velutipes Mycelium. Am. J. Chin. Med. 38, 191. 10.1142/S0192415X10007750 20128054

[B121] Young J. N. Elgash M. (2022). Flagellate Mushroom Dermatitis. JAMA dermatol. 10.1001/jamadermatol.2022.0605 35442408

[B122] Yuan S. Gopal J. V. Ren S. Chen L. Liu L. Gao Z. (2020). European Journal of Medicinal Chemistry, 202. 10.1016/j.ejmech.2020.112502 Anticancer Fungal Natural Products: Mechanisms of Action and Biosynthesis 32652407

[B123] Zaidman B. Z. Yassin M. Mahajna J. Wasser S. P. (2005). Medicinal Mushroom Modulators of Molecular Targets as Cancer Therapeutics. Appl. Microbiol. Biotechnol. 67 (4), 453–468. 10.1007/s00253-004-1787-z 15726350

[B124] Zhang G. Sun J. Wang H. Ng T. B. (2010). First Isolation and Characterization of a Novel Lectin with Potent Antitumor Activity from a Russula Mushroom. Phytomedicine 17 (10), 775–781. 10.1016/j.phymed.2010.02.001 20378319

[B125] Zhang M. Cui S. W. Cheung P. C. K. Wang Q. (2007). Antitumor Polysaccharides from Mushrooms: a Review on Their Isolation Process, Structural Characteristics and Antitumor Activity. Trends Food Sci. Technol. 18 (1), 4–19. 10.1016/j.tifs.2006.07.013

[B126] Zhao S. Gao Q. Rong C. Wang S. Zhao Z. Liu Y. (2020). Immunomodulatory Effects of Edible and Medicinal Mushrooms and Their Bioactive Immunoregulatory Products. J. Fungi 6 (4), 1–37. 10.3390/jof6040269 PMC7712035 33171663

[B127] Zheng S. Li C. Ng T. B. Wang H. X. (2007). A Lectin with Mitogenic Activity from the Edible Wild Mushroom Boletus Edulis. Process Biochem. 42 (12), 1620–1624. 10.1016/j.procbio.2007.09.004

